# Late Brain Involvement after Neonatal Immune Activation

**DOI:** 10.1155/2019/9573248

**Published:** 2019-08-05

**Authors:** Paula Dias, Viviane Freiberger, Letícia Ventura, Daiane Bragagnolo, Matheus L. Dutra, Verônica V. Horewicz, Clarissa M. Comim

**Affiliations:** Research Group on Neurodevelopment of Childhood and Adolescence, Laboratory of Experimental Neuroscience, Postgraduate Program in Health Sciences, University of South Santa Catarina, Palhoça, SC, Brazil

## Abstract

The neonatal immune system is still immature, which makes it more susceptible to the infectious agents. Neonatal immune activation is associated with increased permeability of the blood-brain barrier, causing an inflammatory cascade in the CNS and altering behavioral and neurochemical parameters. One of the hypotheses that has been studied is that neuroinflammation may be involved in neurodegenerative processes, such as Alzheimer's disease (AD). We evaluate visuospatial memory, cytokines levels, and the expression of tau and GSK-3*β* proteins in hippocampus and cortex of animals exposed to neonatal endotoxemia. C57BL/6 mice aging two days received a single injection of subcutaneous lipopolysaccharide (LPS). At 60,120, and 180 days of age, visual-spatial memory was evaluated and the hippocampus and cortex were dissected to evaluate the cytokines levels and expression of tau and GSK-3*β* proteins. The animals exposed to LPS in the neonatal period present with visuospatial memory impairment at 120 and 180 days of age. Here there was an increase of TNF-*α* and IL-1*β* levels in the hippocampus and cortex only at 60 days of age. Here there was an increase in the expression of GSK-3*β* in hippocampus of the animals at 60, 120, and 180 days of age. In the cortex, this increase occurred in the 120 and 180 days of age. Tau protein expression was high in hippocampus and cortex at 120 days of age and in hippocampus at 180 days of age. The data observed show that neonatal immune activation may be associated with visuospatial memory impairment, neuroinflammation, and increased expression of GSK-3*β* and Tau proteins in the long term.

## 1. Introduction

The neonatal period has the highest lifetime risk of serious infections. Neonatal sepsis is defined as a systemic inflammatory response, occurring in the first four weeks of life as a result of a suspected or proven infection. Sepsis is a leading cause of morbidity and mortality in newborn and preterm infants [1–3]. In this context, infections caused by Gram-negative bacteria are of high prevalence during prenatal and neonatal periods. The lipopolysaccharide (LPS) is the major virulent constituent of the outer membrane of Gram-negative bacteria and neonatal LPS exposition seemingly mimics physiological and behavioral alterations triggered by a Gram-negative bacterial infection [4].

The brain of neonates is more vulnerable to damage in response to systemic inflammation. The systemic proinflammatory cytokines is able to increase blood-brain barrier (BBB) permeability and lead to microglial activation. The immunological implications of brain immaturity, particularly with regard to the immaturity of central nervous system (CNS) immune cell regulation, might render brains especially vulnerable to damage by poorly controlled and pervasive inflammation [5, 6].

Studies have been demonstrated that neonatal immune activation by LPS in the early life periods can be associated with short- and long-term consequences. Animals that received a single injection of systemic LPS on the seventh postnatal day showed an increase in apoptotic cells within 24 hours and an increase in IL-6, IL-1*β*, and TNF-*α* levels between 6 and 48 h in brain tissue. Microglial activation was observed after 48 hours of exposure to LPS. These changes persisted up to 7 days after endotoxemia [7]. In the long term, it was reported that systemic immune activation by LPS on the third and fifth postnatal days increased levels of TNF-*α* and IL-1*β* in hippocampus of adult animals [8]. Cognitive impairment also was reported. Comim and collaborates (2016) [9] showed that animals exposed to LPS on the second postnatal days and evaluated when completed 60 days old presented with habituation and aversive and recognition memory impairment. A clinical study by Stoll and collaborates (2004) [10] observed the association between neonatal sepsis and cognitive deficits in children evaluated between 18 and 22 months old. Long-term alterations in brain development following being exposed to LPS result from the fact that neuronal migration, gliogenesis, and myelinogenesis occur at a late gestational age and predominate in the first two weeks of postnatal life [11].

This long-term impairment in brain function was suggested to result from neurodegenerative or ischemic mechanisms triggered by systemic inflammation [12, 13]. In the neurodegenerative processes the diagnosis is generally performed only in advanced or late stages, when brain function is impaired and the treatment is almost ineffective. Alzheimer's disease (AD), one of the most studied neurodegenerative diseases, is associated with an intense neuronal death occurrence, progressive formation of neurofibrillary tangles, and amyloid plaques. These neurofibrillary tangles are formed due to hyperphosphorylation of the protein tau, while amyloid plaques are synthesized by hydrophobic aggregates of misfolded amyloid-*β* peptide (A*β*) [14–16].

Studies showed that alterations in the GSK-3*β* protein expression are also associated with an increase of production and deposition of the A*β* protein in hippocampus [17] and frontal cortex [18] of AD patients. One of the functions of the GSK-3*β* protein is to regulate the phosphorylation of the tau protein. In this context, the increase of GSK-3*β* and tau expression may be involved in AD symptoms, including cognitive deficits [19]. Recently, it has been shown that mechanisms such as chronic neuroinflammation can occur before the classic pathological alterations mentioned above during AD [20]. The hypotheses of this study are that neuroinflammation in the early stages of life may be involved in neurodegenerative processes, such as AD. Thus, the objective of this study is to evaluate visuospatial memory, cytokines levels, and the expression of tau and GSK-3*β* proteins in hippocampus and cortex of animals exposed to neonatal endotoxemia.

## 2. Materials and Methods

### 2.1. Animals

Neonatal male C57BL/6 mice aged 2–3 postnatal days from our breeding colony were used for the experiments. All procedures were approved by the Animal Care and Experimentation Committee of UNISUL 17.003.4.01.IV, Brazil, and were in accordance with the National Institute of Health Guide for the Care and Use of Laboratory Animals (NIH publication no. 80-23), revised in 1996.

### 2.2. Animal Model of Endotoxemia

The technique consists of a single subcutaneous administration of 25 *μ*g/kg of LPS (O26:B6* E. coli* LPS, Sigma Chemical). The control group received a subcutaneous injection of PBS as a placebo in equivalent volume. The animals were monitored on the days following exposure to endotoxemia evaluated to mortality, resulting in survival of 25% after 80 h [9]. The literature reports that this experimental model is the one that most closely resembles neonatal sepsis, since there is a 60% mortality in the first days of life. Eighty percent of survivor animals showed a decrease of body weight and developmental delay until 60 days of age when compared to control animals that received PBS. After the inoculation, the animals returned to their cages and stayed with their mothers until 21 postnatal days. After that, they were separated into five animals per cage until 60, 120, and 180 days old. Each time, the animals were submitted to the behavioral tests and after that it was euthanized and the hippocampus and cortex were immediately isolated on dry ice and stored at −80°C for posterior analysis [9, 21].

### 2.3. Behavioral Tasks

To evaluate the behavioral response, the animals were separated into two groups: control and LPS (n=8 per group and 16 for each time task, n=32). Twenty-one days after inoculation, the animals were randomized and subjected to the Morris water maze test. Thus, using this design, we did not assess time-dependent memory, but memory over time (with new training at each test session). All behavioral procedures were conducted between 08:00 and 10:00 a.m. in a sound-isolated room, and a single animal performed only one behavior test session in only one time point. The behavioral test was recorded by the same person who was blind to the animal group.

### 2.4. Morris Water Maze

The Morris water maze aims to evaluate learning and the ability to acquire spatial memory using environmental tips. The animal should learn how to use the tips attached to the wall of the room to navigate to the submerged platform. During training, the latency is measured for seconds to find the submerged platform (10 cm2). The time the animal has to find the platform, during which each time it is put into the water, is 60 seconds. If the animal does not find the platform, it is gently led up to it; once it is on the platform, it stays on it for 10 seconds. The training takes place in the two days before the test, consisting of six batteries. At the time of testing, the platform is removed, and the animal is put into the water only once; during the three-minute period it will be free to swim. In the test, the time spent in the quadrant in which the platform was located is evaluated. To perform this test, a circular tank located in the center of a room of 12 m2 with an upper window at the south point was used, and the training and tests were performed from 9 o'clock in the morning. To carry out the training, water was added to the tank until the water level exceeded 2 cm of the platform height. Then, the water was left opaque with addition of corn starch, thus making the platform visibility difficult. The acquisition of visual-spatial memory was evaluated after the end of the test through filming and analyzed by the system Any-Maze ® [22, 23].

### 2.5. GSK-3*β* and Tau Protein Expression

Brain tissue samples were manually homogenized with micropistils in ice-cold RIPA buffer containing 1% protease inhibitor (Sigma-Aldrich, St Louis, MO) and then incubated on ice for 30 min. The tubes containing the lysates were centrifuged at 10,000 g for 20 min at 4°C, and the supernatants were collected. Protein concentration was determined using the Bradford method.

The electrophoretic separation was conducted using 30 *μ*g of protein per well in 10% polyacrylamide gel electrophoresis (SDS-PAGE), running in a Mini-PROTEAN® Tetra cell apparatus under a PowerPAC™ HC power supply (both from Bio-Rad, CA, USA). The proteins were transferred onto a PVDF membrane (Bio-Rad Laboratories Inc., Hercules, CA, USA), blocked in 5% BSA (prepared in TBS-T buffer, pH 7.4; concentration in mmol/L: 20 Tris-HCl, 137 NaCl, 0.1% Tween 20), and incubated overnight at 4°C with primary antibodies to GSK-3*β* (1:1000, Cell Signaling Technology, Beverly, MA, EUA) and TAU (1:1000, Cell Signaling Technology, Beverly, MA, EUA). Peroxidase-conjugated monoclonal antibody against *β*-actin (dilution 1:45000) was used as a loading control for all samples tested. After incubation with primary antibodies, the membranes were washed three times (10 minutes each) with TBS-T solution and incubated with the specific secondary antibody conjugated to horseradish peroxidase (HRP) at room temperature for 1 h. The membranes were washed for another three times (10 minutes each) with TBS-T solution and exposed to HRP substrate (Pierce Biotechnology, Rockford, IL, USA), and immune complexes were visualized by chemiluminescence using ChemiDoc MP System (Bio-Rad Laboratories). Bands were quantified by densitometry using the software from the manufacturer (Image Lab, version 4.1, Bio-Rad Laboratories, Hercules, CA, USA). Values were normalized using the data obtained for *β*-actin and expressed as arbitrary units.

### 2.6. Cytokines Levels

The concentration of cytokines (IL-1*β* and TNF-*α*) was determined by ELISA (R&D Systems, Minneapolis, MN). All samples were assayed in duplicate. Briefly, the capture antibody (13 mL, contains 0.1% sodium azide) was diluted in phosphate-buffered saline (PBS), added to each well and left overnight at 4°C. The plate was washed four times with PBS and 0.05% Tween 20 (Sigma, St. Louis, MO, USA). The plate was blocked with 1% bovine serum albumin and incubated for 1h at room temperature before washing four times with PBS and 0.05% Tween 20. The samples and standards were added, and the plate was incubated overnight at 4°C. After washing the plate, detection antibody (concentration provided by the manufacturer) diluted in PBS was added. The plate was incubated for 2h at room temperature. After washing the plate, streptavidin (DuoSet R&D Systems, Minneapolis, MN, USA) was added and the plate was incubated for 30 min. At last, color reagent o-phenylenediamine (Sigma, St. Louis, MO, USA) was added to each well and the reaction was allowed to develop in the dark for 15 min. The reaction stopped with the addition of 1 M sulfuric acid to each well. The absorbance was read on a plate reader at 492 nm wavelength (EMax, Molecular Devices, Minneapolis, MN, USA). Total protein was measured using bovine serum albumin as a standard as described in the literature [24].

### 2.7. Statistical Analyses

Shapiro-Wilk normality test were utilized to determine the parametric and nonparametric data. Data from the behavioral test and biochemical analyses are parametric data and it were reported as mean±SEM and analyzed by Student's t test. A ^*∗*^p < 0.05 was considered statistically significant.

## 3. Results

**Figure 1:**
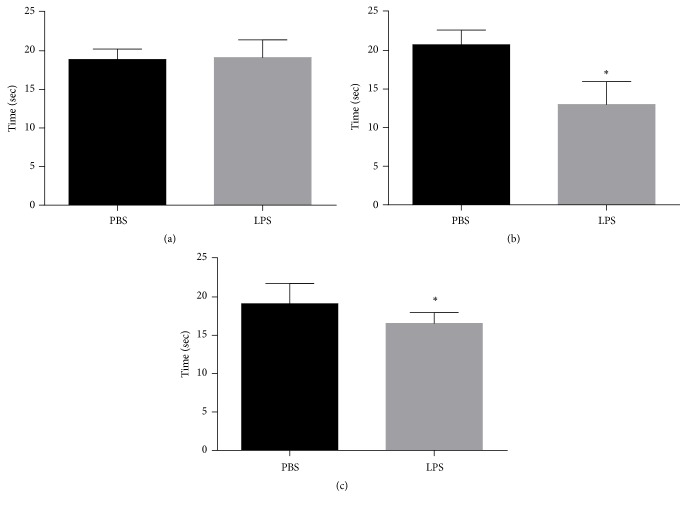
Morris water maze. (a, b, c) Demonstrating the results for 60, 120, and 180 days after neonatal immune activation, respectively. Data are expressed as mean and standard deviation. ^*∗*^p <0.05 versus PBS. n = 8.

Figure 1 shows the results obtained in the Morris water maze test. The dwell time in the quadrant where the platform was located was evaluated in the test session. It was observed that after 60 days (Figure 1(a)) of endotoxemia, there was no statistically significant difference between the groups evaluated (p = 0.794). However, after 120 (Figure 1(b)) and 180 (Figure 1(c)) days, a significant decrease in the residence time in the quadrant where the platform was located (p <0.0001 and p = 0.0403, resp.) was observed.

**Figure 2:**
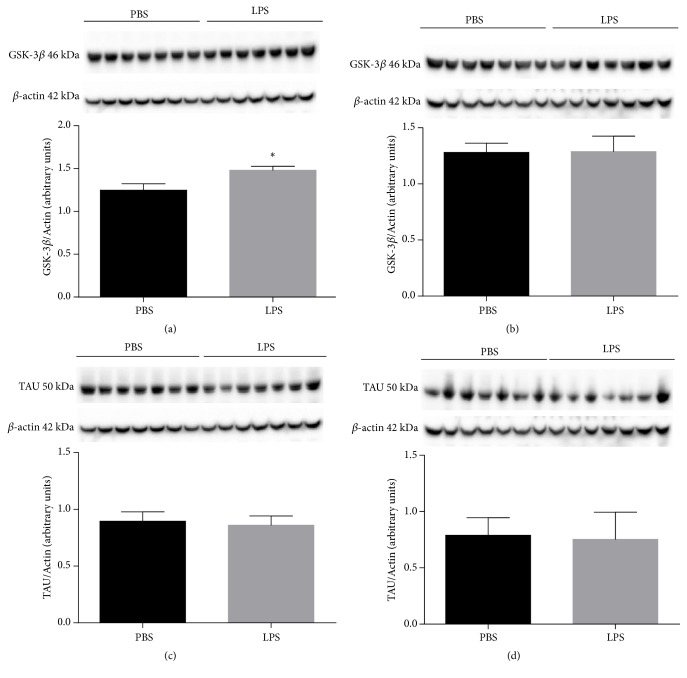
Expression of GSK-3*β* protein in hippocampus (a) and cortex (b) and Tau protein in hippocampus (c) and cortex (d) of animals 60 days after neonatal immune activation. Data are expressed as mean and standard deviation. ^*∗*^p <0.05 versus PBS. n = 8.

**Figure 3:**
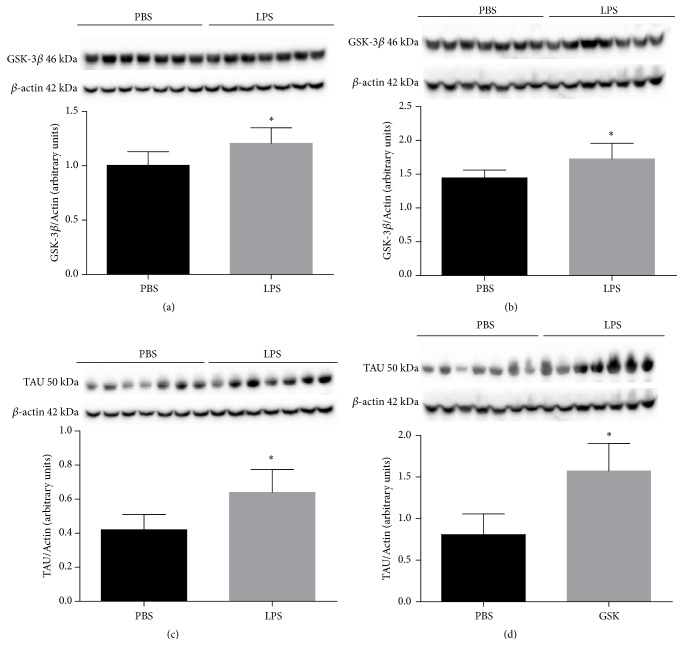
Expression of GSK-3*β* protein in the hippocampus (a) and cortex (b) and Tau protein in the hippocampus (c) and cortex (d) of animals 120 days after neonatal immune activation. Data are expressed as mean and standard deviation. ^*∗*^p <0.05 versus PBS. n = 8.

Figure 2 shows the results of GSK-3*β* protein expression in the hippocampus (Figure 2(a)) and cortex (Figure 2(b)) and TAU in the hippocampus (Figure 2(c)) and cortex (Figure 2(d)) in animals receiving LPS or PBS at 2 days of age and was evaluated when they completed 60 days old. It was observed that after 60 days of endotoxemia there was a significant increase in the expression of the GSK-3*β* protein in the hippocampus of animals receiving LPS when compared to animals receiving PBS (p <0.0001, Figure 2(a)). However, there were no statistically significant alterations in the evaluations of GSK-3*β* protein expression in hippocampus (Figure 2(b)) and TAU protein in hippocampus (Figure 2(c)) and cortex (Figure 3(d)) (p> 0.05) after 60 days of endotoxemia.

Figure 3 expresses the results of GSK-3*β* protein expression in the hippocampus (Figure 3(a)) and the cortex (Figure 3(b)) and TAU in the hippocampus (Figure 3(c)) and cortex (Figure 3(d)) in animals receiving LPS or PBS at 2 days of age and was evaluated when they completed 120 days old. It was observed that there was a statistically significant increase in GSK-3*β* protein expression in the hippocampus (Figure 3(a), p = 0.0150) and in cortex (Figure 3(b), p = 0.0396) when compared to the group of animals receiving PBS. There was also a significant increase in TAU protein expression in hippocampus (Figure 3(c), p = 0.0127) and in cortex (Figure 3(d), p = 0.0004) compared to the PBS group at 2 days old.

**Figure 4:**
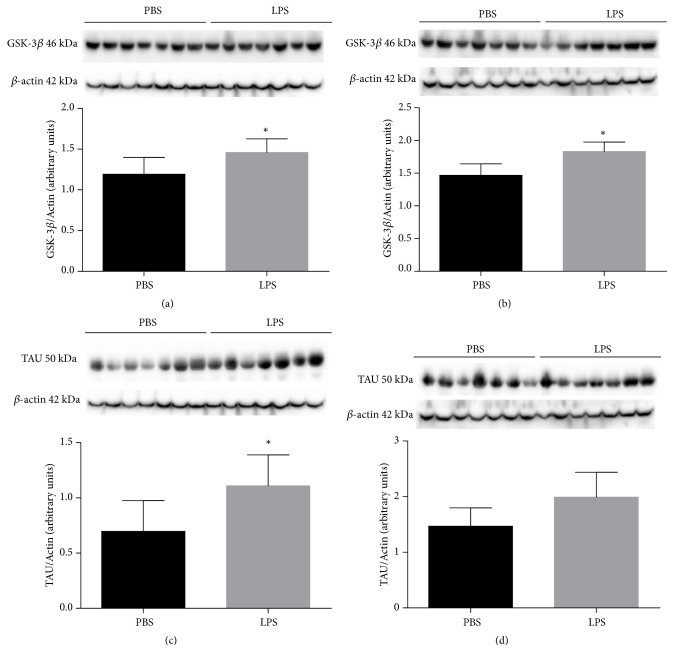
Expression of GSK-3*β* protein in hippocampus (a) and cortex (b) and Tau protein in hippocampus (c) and cortex (d) of animals 180 days after neonatal immune activation. Data are expressed as mean and standard deviation. ^*∗*^p <0.05 versus PBS. n = 8.

Figure 4 expresses the results of GSK-3*β* protein expression in the hippocampus (Figure 4(a)) and the cortex (Figure 4(b)) and TAU in the hippocampus (Figure 4(c)) and cortex (Figure 4(d)) in animals exposed to LPS or PBS at 2 days and was evaluated when they completed 180 days old. It was observed that there was a statistically significant increase in GSK-3*β* protein expression in hippocampus (Figure 4(a), p = 0.040) and in cortex (Figure 4(b), p = 0.0066) of the animals exposed to LPS when compared to the group of animals which received PBS. As for TAU protein, there was a significant increase in hippocampal expression (Figure 4(c), p = 0.0171). However, in the cortex (Figure 4(d)), there was no significant difference between the groups (p = 0.0680).

**Figure 5:**
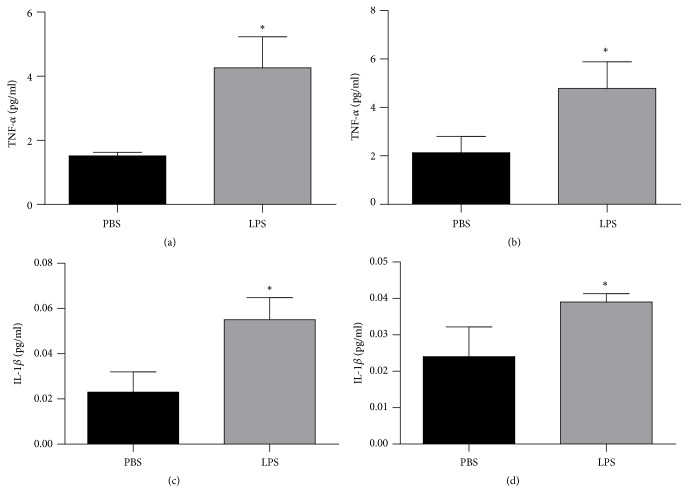
Levels of TNF-*α* hippocampus (a) and cortex (b) and IL-1*β* in hippocampus (c) and cortex (d) of animals 60 days after neonatal immune activation. Data are expressed as mean and standard deviation. ^*∗*^p <0.05 versus PBS. n = 8.

The cytokines levels were demonstrated in Figure 5. These levels were evaluated through the levels of TNF-*α* in hippocampus (Figure 5(a)) and cortex (Figure 5(b)) and IL-1*β* in hippocampus (Figure 5(c)) and cortex (Figure 5(d)) in animals exposed to LPS or PBS at 2 days and were evaluated when they completed 60 days old. There was an increase of TNF-*α* in hippocampus (Figure 5(a), p = 0.0182) and (Figure 5(b), p = 0.0238) and IL-1*β* in hippocampus (Figure 5(c), p = 0.0376) and cortex (Figure 5(c), p = 0.0012) when compared with PBS group.

**Figure 6:**
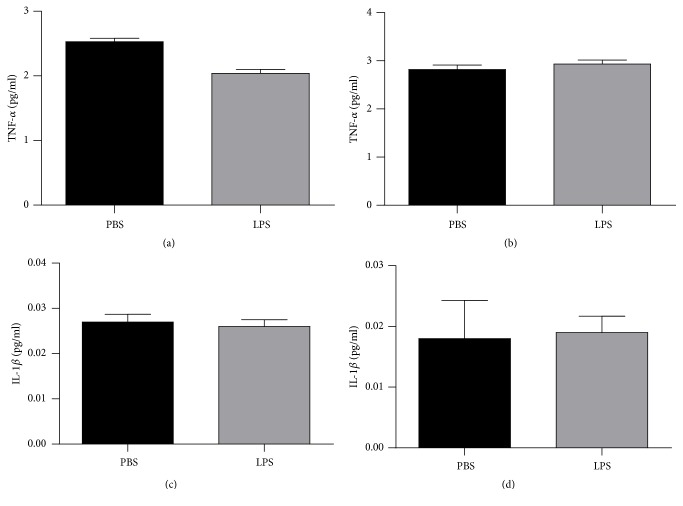
Levels of TNF-*α* hippocampus (a) and cortex (b) and IL-1*β* in hippocampus (c) and cortex (d) of animals 120 days after neonatal immune activation. Data are expressed as mean and standard deviation. n = 8.

Figure 6 demonstrated the results of TNF-*α* in the hippocampus (Figure 6(a)) and the cortex (Figure 6(b)) and IL-1*β* in the hippocampus (Figure 6(c)) and cortex (Figure 6(d)) in animals exposed to LPS or PBS at 2 days and were evaluated when they completed 120 days old. There were no significant differences between groups in the TNF-*α* and IL-1*β* in the evaluated structures (p>0.05).

**Figure 7:**
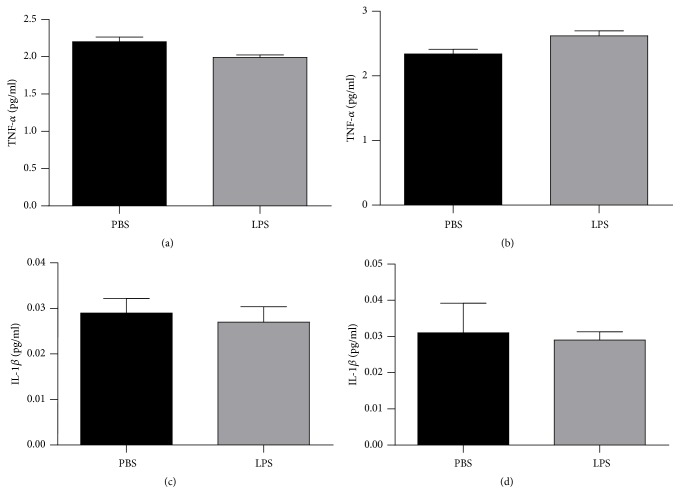
Levels of TNF-*α* hippocampus (a) and cortex (b) and IL-1*β* in hippocampus (c) and cortex (d) of animals 180 days after neonatal immune activation. Data are expressed as mean and standard deviation. n = 8.

Finally, Figure 7 showed the results of TNF-*α* in the hippocampus (Figure 7(a)) and the cortex (Figure 7(b)) and IL-1*β* in the hippocampus (Figure 7(c)) and cortex (Figure 7(d)) in animals exposed to LPS or PBS at 2 days and were evaluated when they completed 180 days old. There were no significant differences between groups in the TNF-*α* and IL-1*β* in the evaluated structures (p>0.05).

## 4. Discussion

The results of this study demonstrate that the animals exposed to LPS in the neonatal period and evaluated at 120 and 180 days old presented with visuospatial memory alteration. As regards protein expression, there was an increase in GSK-3*β* expression in hippocampus when the animals completed 60 days old. In animals with 120 days of age, there was an increase in GSK-3*β* and Tau expression in hippocampus and cortex. In animals with 180 days of age, there was a significant increase in GSK-3*β* protein expression in hippocampus and cortex and Tau protein only in hippocampus. There was also an observed increase of TNF-*α* and IL-1*β* levels in the hippocampus and cortex only at 60 days old.

The LPS entry into the brain after systemic administration is in small amounts; the increased expression of proinflammatory mediators is capable of increasing the permeability of the blood-brain barrier leading to a microglial activation. Once activated, microglia initiate a neuroinflammation process sustained by the release of proinflammatory cytokines directly into the brain tissue, intensifying damage to neuronal and glial cells [6, 25–27]. Microglia in the neonatal period are responsible for a process known as synaptic pruning, directly influencing synaptogenesis. Microglial dysfunctions in the early postnatal days are associated with cerebellar hypoplasia, neuronal loss and retraction, delay in the myelination process associated with changes in progenitor cell proliferation and differentiation, and increased expression of TLR-4 receptors and matrix myeloperoxidase 9 (MMP-9) and astrogliosis in brain tissue a few days after exposure to LPS in an animal model [28].

In a long term, the endotoxemia in the early life periods is associated with cognitive impairment in animal model [9] and patients [10]. The cognitive alterations are correlated to increase TNF-*α* and IL-1 in hippocampus [8]. In addition, cytokine overexpression has been associated with neuropsychiatric diseases such as depression [29–32] and neurodegenerative diseases such as AD and other dementias [33, 34]. Long-term alterations in brain development following LPS-induced neonatal immune activation result from the fact that neuronal migration, gliogenesis, and myelinogenesis occur at a late gestational age and predominate in the first two weeks of postnatal life [11]. In addition, it was demonstrated that a single systemic injection of LPS on the fourteenth postnatal day altered social behavior when the animals aged 21 days [35].

In addition to the behavioral consequences described above, pre- and postnatal exposure to LPS in rodents also include autism-like behaviors induced by prenatal exposure on the ninth embryonic day [36], schizophrenia-like behaviors induced by LPS exposure on the 15th embryonic day [37], and anxiety-like behaviors induced by LPS exposure on the third and fifth postnatal days [8]. However, on the long-term consequences related to neurodegeneration, there are few reports in the literature.

Evidence suggests that the neuroinflammation processes are involved in the development of AD [38–40] and TNF-*α* plays an important role during the inflammatory response. The results of the present study demonstrated that animals exposed to endotoxemia in the neonatal period presented with increased levels of cytokines, TNF-*α*, and IL-1*β* in the hippocampus and cortex at 60 days old. In cross-sectional studies, these cytokines have been associated with cognitive deficits and dementia [41–43] and in clinical cohort studies in AD [39, 40]. As in studies with transgenic animals with AD, elevated TNF-*α* levels were observed in the brain tissues of these animals [44].

In the present study, it was possible to observe that endotoxemia in the neonatal period altered visuospatial memory only when the animals completed 120 and 180 days old. After 60 days, when the mice were considered young adults, there was no alteration of the visuospatial memory. This memory consists of the ability to encode, store, and retrieve information about spatial locations. The hippocampus and cortex are areas involved in the formation and storage of visual-spatial memory. Deficits in visuospatial memory are commonly observed in subjects diagnosed with neurodegenerative diseases, such as AD.

The AD is characterized by a progressive loss of visuospatial abilities associated with an accumulation of senile plaques, neurofibrillary tangles, and neuronal loss mainly in the hippocampus and temporal cortex [45, 46]. In addition to the changes described above, another important feature in the degenerative process of AD is the increased expression of GSK-3*β* and Tau proteins in brain tissue. Studies have shown that alterations in the GSK-3*β* protein are also associated with increased production and deposition of the A*β* protein. In this study, there was an increase in the expression of the GSK-3*β* protein in hippocampus of animals with 60, 120, and 180 days of age. In cortex, GSK-3*β* expression increased only in animals at 120 and 180 days of age. Visuospatial memory alteration was also observed only in animals with 120 and 180 days of age.

One of the functions of the GSK-3*β* is to regulate the phosphorylation of the Tau. Hyperphosphorylated Tau was found mainly in hippocampus and temporal lobe regions during the pathophysiological process of AD. Increased expression of GSK-3*β*, Tau, and other substrates may be involved in AD symptoms, including cognitive deficits [18]. Changes in GSK-3*β* and Tau proteins are related to axonal transport, leading to impaired memory and learning as a result of synaptic dysfunction [18, 47, 48].

After the above explanation, increased expression of GSK-3*β* and Tau proteins in regions highly involved in learning and memory could be associated with impairment of visuospatial abilities. A consistent finding observed in this study was that, in animals at 60 days of age, increased GSK-3*β* expression was observed in the hippocampus associated with increased cytokines levels in hippocampus and cortex. However, these animals did not show visual-spatial memory alteration. Alteration in visuospatial memory was only demonstrated in animals at 120 days of age, that is, when there was an increase in the expression of GSK-3*β* and Tau proteins, both in the hippocampus and in the cortex. At 180 days old, the animals exposed to LPS in the neonatal period still presented with alterations in visuospatial memory associated with an increase in the expression of GSK-3*β* and Tau in hippocampus and only GSK-3*β* in cortex. In this time, we did not observe increase of cytokines levels.

## 5. Conclusion

In this context, it is believed that the results of this study may contribute to strengthening the evidence that a process of systemic neonatal immune activation can cause a change in visual-spatial memory and increase the expression of GSK-3*β* and Tau proteins in hippocampus and cortex in later periods.

## Data Availability

The data used to support the findings of this study are available from the corresponding author upon request.
